# Science, Sentiment, and the State: Community Genetics and Pursuit of Public Health in Cuba

**DOI:** 10.1111/maq.12056

**Published:** 2013-11-08

**Authors:** Sahra Elizabeth Gibbon

**Affiliations:** Department of Anthropology, University CollegeLondon

**Keywords:** genomics, public health, Cuba, health professionals

## Abstract

Contributing to an emerging field of social science literature by examining the translation of genomic medicine across global and transnational fields of research and medicine, this article examines how genetics is allied to public health in Cuba. It examines the sociopolitical and cultural discourses and practices that constitute community genetics or challenge or impede the translation and expansion of genomics as public health. Focusing on the experience of health practitioners, the article explores how their work is circumscribed by cultural values and social ideologies that collectively reveal an unexpected heterogeneity in how genetics is being constituted and reproduced. Although the Western quest for genomics as “personal medicine” is revealed here as both ideologically and practically problematic, such challenges paradoxically work to reinforce a commitment to maintaining the distinctive field of Cuban community genetics in its orientation to collective public health.

## Field Note, Havana April 2006

It is the third day of a five-day conference where nearly 600 medical and scientific personnel, made up of a cohort of mostly newly trained health professionals who have undertaken their masters in genetic counselling have gathered for the first national meeting of Cuban Community Genetics. There is more than an edge of excitement in the air, in part because for many it is the first time they have stayed in one of the Western-style hotels on the edge of Havana near the conference location. But there is also a rumor that “Fidel will visit.” At 5pm, at the end of a full third day of workshops and presentations, we are instructed to enter the large conference hall whose 3,000 seats are now filled by various dignitaries and young *trabajadores sociales* (community social workers). Before finding out if indeed the rumor is true, a variety of different entertainments commence.

First, a skilled display of ballet by a group of children with learning difficulties is performed in front of a huge banner flung across the stage that says *Por La Vida* [For Life]. The group of female geneticists sitting to my right are visibly emotionally affected by the display. Later I learn that this is because they know personally both the families and the children who have performed. The emotive weight is increased for many by the screening following this performance of a documentary film about how Cuban medical professionals treated children from the Chernobyl disaster in the early 1990s. One of the male biologists I am sitting alongside has tears in his eyes and acknowledges unashamedly how impressed and moved he is by the work of these professionals. Sometime soon after this, the “rumor” that had been circulating earlier proves to be true and Fidel Castro and his entourage duly arrive to what seems both orchestrated and genuine wonder.

The profile, attention, and resources that are being targeted at the development of genetics and the way this is being nested within the programs of Cuban public health becomes apparent during the course of the lengthy evening that unfolds. It is conveyed by the impressive and exhausting six-hour speech given by Castro, subsequently broadcast as a series of televised evening news programs, in which achievements in Cuban biotechnology, including vaccines for conditions such as meningitis and cochlear implant technology for children, are used to highlight the collective public health dimensions of these innovations. The seemingly on-the-hoof decision, personally announced by Castro, to give not only computers but Internet access to 500 newly trained genetic health professionals in order to maintain their ability to access cutting-edge research delights many of those I'm sitting with. It's a decision that seems to give gusto to the final singing of socialist anthem “The Internationale” at the end of the very long day and evening.

The first national meeting of *Genética Communitaria* (Community Genetics) was something of a watershed moment in a highly political conjoining of medical genetics and public health as part of an ongoing socialist project of Cuban health care. The emphasis on the “ethical” development of biotechnology, and the promise of home computer use and Internet access to individual genetic health professionals, coupled with the emotive representations of Cuban doctors and the children's dance performances, underlaid the diverse registers in which medical genetics was being represented and reproduced.

A focus on community genetics is certainly not unique to Cuba^1^ and has, in fact, only been formally established there since 2004. Nevertheless, there are currently over 1,600 persons employed nationally in this field as genetic specialists, technicians, counselors, and nurses. They form part of a state organization and regionally based national network of genetic centers and clinics that conduct over “40,000 diagnostic tests per year” (Teruel 2009:11). The working structure of Cuban community genetics directly mirrors the system of *consultorios* and clinics established as part of the family medicine initiative in the 1980s. With over 184 genetic centers spread across all Cuban municipalities, community genetics has evolved over the last five years, with the stated aim of developing “a national programme for the diagnosis, management and prevention of genetic diseases and congenital malformations” (Teruel 2009:11).

The eclectic and heterogeneous ways in which this area of health care was articulated during the event described above serves as a starting point for exploring the cultural meanings and sociopolitical significance of medical genetics as public health in Cuba. Drawing on fieldwork with genetic health professionals who work and live in their local community, this article examines the ways in which medical genetics is constituted and practiced as public health in a context where (for now at least) existing state socialism colors public and professional engagement. It examines how, on the one hand, in a context of scarce resources, state discourse about concerns with collective health are marshalled, often in opposition to and in explicit contrast to what is rhetorically seen as “capitalist individualism.” At the same time, a seemingly more Western vision of technologically driven genomics as promissory health care and/or “personalized medicine” is reconfigured as part of program of socialist health care (see, e.g., Abu el Haj 2007).

Rather than arguing that clear distinctions can be drawn between assumed paradigmatic political forms of genetic health care (e.g., personalized or collective, with attendant differences in modes of governance and practice), this article explores how both novel *and* preexisting discourses and practices relating to genetics and public health make Cuban community genetics possible. It illustrates how the translation of genetic health must be understood at these intersections, revealing the challenges or impediments to those efforts and the local registers that inform how genetic medicine as public health is practiced and reproduced. In this way, the case is made for understanding the transnational expansion of genetic health care as heterogeneous rather than singular or uniform.

## The Biopolitical Rationalities of Genomic Health in Global Perspective

The notion that developments in medical genetics have been and are bound up with particular kinds of politics or modes of governance have long formed a central pillar in the cultural critique and commentary of social scientists examining these developments (Clarke et al. 2003; Rose 2001). It has been argued that an era of genomics is necessarily one that is concerned with questions of consumer or patient rights, liberties, and autonomy such that the demands and promises of new genetic knowledges or technologies necessarily ensure access to and/or obligations to engage with specific regimes of “(self) care.” For Rose and Novas, genetic medicine operates in a political and ethical field in which “a prudent yet enterprising individual [is] actively shaping his or her life course through acts of choice” (Rose and Novas 2005:458). An ongoing emphasis on the goal of tailored or personal medicine points to the persistence of such a biopolitical rationality in what has been described as a “post-genomic era” (Sunder-Rajan 2006:20). To a great extent, the politics behind the promissory goal of genetic medicine continue to be influential even as it becomes coterminous with more hesitant rhetoric about complexity and the (as yet) unfulfilled promise of genomics. Nadia Abu el Haj, for example, notes that “post-genomic medicine [still] operates in a neo-liberal economy characterized by re-entrenchments of the welfare state, the de-regulation of industry and the privatization of biological research” (2007:290).

Recent social science studies in European and American societies, however, have raised questions about an approach that assumes and has tended to focus on cultures of activism and the reformulation of individual and collective subjectivities in relation to expanding fields of genetic health care, of a “biopolitics from below.” Such work calls for rethinking how “the molecular, the population and the life sciences are in linked in more complicated ways” (Raman and Tutton 2009:21). Attending to the ongoing role of the state in public health interventions related to genetics and the variable ways patient communities may be indifferent to, actively reject, or be excluded from the possibility of engagement with genomic health (Plows and Boddington 2006) are important correctives in this regard. What is important here is recognizing “that there is not a singular politics of life, but multiple politics with inequalities, opportunities, complexities and dilemmas, both individual and collective, which require a more nuanced exploration” (Raman and Tutton 2009:20).

Comparative social science studies outside of Europe, Canada, and the United States highlight the importance of considering how diverse forms of governance are instrumental to research and how medical practices are linked to novel developments and opportunities in the life sciences. For some, a global perspective illuminates inequities in the provision of and/or access to new health technologies (Biehl 2007; Nugyen 2005). Others illustrate how “bio-available” populations become constitutive of genomics as transnationally connected developments in the life sciences, including clinical trial research, extend to include and/or become reliant on populations in resource poor communities (Bharadwaj 2008; Petryna 2007; Sunder-Rajan 2006). For others, a global comparative perspective is about understanding how the vitality of expanding areas of genomic knowledge and health care is directly connected to state interventions and interests. In these contexts, assuming that self-actualizing, individual neoliberal identities and identifications are always necessary prerequisites to and/or a direct consequence of novel medical techniques misapprehends broader historical and political dynamics. As Susan Greenhaulgh puts it:

When we consider the rest of the world, which includes 4/5s of the global population, the rising global powers of China and India, with their very different historical and political rationalities, their more collectivist mentalities and the ongoing reorganization of power at transnational and global levels—a different conclusion seems warranted. [2009:207]

Recent studies examining the dynamics between public health and genomics in Asia including China, Vietnam, Korea, makes a powerful case for reconsidering how not only the long history of state interventions in public health may influence current developments in genomic medicine but also how the old and new may be conjoined in unexpectedly productive ways. Here, as Wahlberg points out, in Vietnam a concern with collective population health or “molar bodies” may be as important as or even conjoined and/or constitutive of an emerging molecular body (2009:242). That is, biopolitical governance may mesh in complex ways with representations of nationhood, the governance of ethics, and sociopolitical histories in ways that do not necessarily *exclude* the possibility of constituting or expressing neoliberal identities. Rather, such expressions intersect or are nested within socioculturally and historically specific modes of identification. What has been described as a “zone of exception,” constituted by the establishment of market zones in the People's Republic of China (Ong 2006), is just one recent example of the complex articulations that are now being explored in the context of biotechnologies and genomic medicine (see Sleeboom-Faulkner 2009).

We need to understand and examine Cuban attempts to incorporate genetics as part of a program of national public health care at the intersections between national or local specificity and the transnational global space of genomic medicine. In this way, we see how Cuban community genetics is a heterogeneously constituted domain of discourses and practices that is emerging in dynamic response to broader global social processes.

I have explored some of these themes in previous discussions about issues of patient subjectivity in relation to Cuban community genetics (Gibbon 2009, 2010). Here, I focus on the practices, experiences, and perspectives of practitioners to examine how policies and practices relating to community genetics and the promise of personalized medicine are managed, enacted, and negotiated by these health professionals. The fieldwork that informs this article was undertaken at various times between 2006 and 2008. My research arose in conjunction with a growing interest in common complex adult onset conditions such as breast cancer by Cuban community genetic practitioners. This developed into a collaborative project to examine health beliefs among a selected group of Cubans about the causes of breast cancer through use of a questionnaire. This article, however, draws from the ethnographic data I collected working with and alongside Cuban community genetic professionals in three different parts of the country, all outside of Havana.

I first outline the emergence and scope of community genetics in Cuba, exploring its relationship to longstanding public health institutions as well as to the experimental milieu of Cuban biotechnology. The multiple registers through which community genetics is being constituted is further illuminated in the narratives of health professionals working in this field and the way they both acknowledge and subsume state paternalism in their daily practice. Yet, community genetics, like other areas of public health in Cuba, is also increasingly and necessarily part of local economies of exchange in a climate where medical supplies and services are scarce, giving new meaning to the provision of state health care as part of local moral worlds of health practice. The final section of the article explores some of the difficulties of fully incorporating breast cancer genetics into Cuban community genetics and the ways these challenges are met and diversely ameliorated by health professionals.

## The Project of Public Health, Science, and Community Genetics

Classified as a developing nation but with a public health program in which the goals of equity and universal access have been and continue to be vociferously championed as part of the history of political struggle that began with the socialist revolution of the late 1950s, the so-called Cuban paradox has long fascinated social commentators. That is how a country with so few economic resources can seemingly maintain statistical health indicators that are comparable to and, in some cases better than, many Euro-American countries. Although these indicators have ensured that public health has long stood as an important symbol of the success of a 50-year socialist project (see Feinsilver 1993), recent studies nevertheless suggest that this profile is being undermined by the effects of the long-running U.S. embargo and the collapse of Soviet subsidies in the 1990s (Andaya 2010).

This situation has highlighted the importance of local social networks of exchange in efforts to sustain health and well-being (Brotherton 2005, 2008). The recent intensification of the export of Cuban medical doctors to neighboring South American countries as part of an extension of a longer standing program of humanitarian medical missions is yet another illustration of the shifting political dynamics of public health that now constitutes health workers as a vital economic component of the Cuban export industry.^2^

Although an ongoing climate of shortage has provoked new economies of informal (and international) exchange or entrepreneurship, Cuba is not (as yet) comparable to China in the opening up of neoliberal markets.^3^ Nonetheless, the country has for some time been competing on a global economic stage in some aspects of biotechnology. The so-called Western scientific pole in Havana employs over 12,000 persons across a range of different centres and facilities, producing vaccines and generic drug products and laying claim to have produced the word's first meningitis B vaccine (Thorsteinsdóttir et al. 2004). The majority of this production is focused on cheap medical technologies aimed at improving *collective* public health interventions. In this sense, Cuba simultaneously reproduces global biotechnology while also helping articulate what might be seen an alternative politics of need in relation to the goals of collective health care.

Simon Reid-Henry's recent examination of Cuban biotechnology highlights how an ostensibly “capitalist form of science” is developing in a socialist country. He points out how this constitutes a “distinctive local experimental milieu” in which health care technologies are “nurtured within a different space of ethics and possibility” that is both aimed at improving public health and facilitating participation in “the patent game” of transnational investment in biotechnology (Reid-Henry 2011:2).

The reference center in Havana for community genetics is officially identified as one of the 50 production and research facilities that make up the “Cuban scientific pole,” illustrating the connections now being forged between public health genetics and biotechnology. Yet, in fact, Genética Communitaria traces its roots to other state interventions in public health, building on a nearly 40-year program of infant and maternal health and the program of family medicine set up in the 1980s. For some, it was the integration of hospitals, local polyclinics, and community-based services as well as a focus on infant and maternal health that enabled Cuba “to realise the principle of ‘health for all’ with a primary focus” (Spiegel and Yassi 2004:97). The training of tens of thousands of medical professionals was also crucial to achieving this goal with many doctors living and working in local consultorios attending to the “physical and social well being” of patients (Nayeri 1995:324).^4^

Given the association with Cuban public health programs, it is perhaps not surprising that the main day-to-day focus of the work of community genetic health professionals relates to neonatal health services. This includes screening newborns for rare chromosomal disorders and congenital malformations and managing national programs of prenatal screening for diseases such as sickle cell anemia and Down's syndrome (Teruel 2009). In a very obvious and immediate sense, medical genetics in Cuba is therefore circumscribed by highly regulated longstanding interventions in the collective management of public health organized and practiced at the local level through the system of family medicine.^5^

Although complex adult onset non-Mendelian conditions are still a minor aspect of day-to-day work, they have begun to become part of the work of community genetics in recent years. The collection of family history information for conditions such as schizophrenia, Alzheimer's, heart disease, diabetes, and a range of common cancers, including breast cancer, have now become part of fledgling efforts in Cuba to “examine the role of genetic factors in these conditions,” with over 43,000 families included on national registries kept since 2004 (Teruel 2009:13). Interest in these conditions is indicative of the country's changing epidemiological disease profile, transformed in recent years from one concerned with diseases of poverty to those of development, including cancer and heart disease.^6^

Somewhat paradoxically, cancer, a disease often perceived as a symbol of modern consumer society (Karakasidou 2008), now has an incidence in Cuba that is comparable to a First World country and has become the focus of Cuba's biotechnology industry aimed at developing cheap and affordable cancer vaccines (Lage 2009). Although the collection of detailed family histories that can be connected to clinical and/or phenotypic data are an increasingly valuable international resources with respect to the future application of genomic medicine (as they can be in Cuba), there are, however, few resources to provide widespread genetic testing, let alone basic screening for those identified as being at risk. Nevertheless, the task of collecting family history data and identifying information relating to persons and families at genetic risk for complex adult onset conditions are an increasing part of the work of community genetic practitioners.

## Being Community Genetic Practitioners in Cuba: A “Revolutionary” Vocation

As the event described in the opening sections of this article suggests, the place of passion and sentiment in the representation of doctors as heroic figures has a longstanding history in the rhetoric of revolutionary Cuba (Fernandez 2000). Given that the image of the self-sacrificing doctor working for the health of the collective population has long provided something of a moral center point for Cuban socialist goals (Feinsilver 1993), it was not surprising to find that this was identifiable in the working environment of community genetics.

Like other state institutions, including hospitals, it was common to see in the entranceway to community genetic clinics hand-made bulletin boards and murals covered not only with relevant information related to infant or maternal health, such as guidance about breast feeding, but also hand-cut-out images of Fidel Castro, Hugo Chavez, and Che Guevara. More striking were what might be described as shrines inside some of the clinics where homage was paid to heroes and heroines of a range of historical revolutionary struggles. In one reception area in a clinic in the southeast of the country, there was a crib decorated with white ribbons and above it the photograph of the wife a 19th-century hero of the first war of Cuban independence. The values relating to female struggle, nurturance, and sacrifice represented in the memorial were complemented on the walls of the clinic with more abstract modernist images of women's pregnant bodies surrounded by tropical flora and fauna. Although these memoralized displays were clearly intended to promote revolutionary values, it was significant that the focus of attention and pride for the health professionals who worked in these settings was often the modern technologies being used, such as the ultrasound scanner.

Community genetic clinics are certainly not unique public spaces for visual or verbal political rhetoric and sloganeering. Still, their presence provides some indication of the way genetic medicine is being incorporated within a collective narrative of revolutionary struggle as part of a socialist public health endeavor. In doing my research alongside genetic health professionals, I could also see how a narrative of heroic national struggle and sacrifice were also aspects of personal narratives, as these excerpts illustrate.

## Excerpt 1

On the first day I meet Maira the director of a regional community genetic clinic in the eastern part of the country she brings her two children to meet me and immediately takes me to the small provincial museum. The history is recounted by the guide of a locale which has been linked to past and current revolutionary conflict in different ways. This includes the War of Independence against Spain and the struggle against slavery in the 19th century. Tales of individual and collective resistance abound. Maira listens intently, despite this being a history she must know intimately. Her rapt attention is partly explained when she tells me that she is distantly related to the most well-known 19th-century revolutionary figure in the region. Over the following weeks, traveling with Maira each day to the clinic through the city, she proudly points out how community genetics has developed in the town with strong links to other areas of public health including the maternity hospital and other clinics linked to diabetes and retinoblastoma.

It is on these journeys that she also tells me her own story of overcoming adversity in her personal life with the unexpected loss of loved ones. This has, she says, helped her in working within community genetics where overcoming tragedy, difficulty, and adversity is common, working with families of children with rare, often fatal, congenital conditions. For Maira, working in community genetics is explained as a kind of fated destiny wherein the personal and political intersect in a region where struggle and sacrifice has long been part of a local identity, commonly identified as the place “where the Cuban revolution first began.”^7^

## Excerpt 2

In a province in the centre of the country, the daily contradictions of life are more stark here than in other places in Cuba where I have been working. Tourist buses whistle through the town on their way to one of the largest beach resorts in the country. In contrast to the frequency of tourist buses, transport to other nearby towns are infrequent, as I and Theresa discover while waiting for hours for a bus to visit one of the nearby genetic clinics. “*No somos as chicas com mucho suerte hoje*” [We're not the girls with much luck today], she jokingly says. It is nevertheless time that provides space for chatting and reflection on some of the contradictions of Cuban life. Seeing the buses laden with tourists going past prompts her to remember the time when she also used to visit the nearby beaches with her family. These were holidays paid for by the state and when access to these beaches was not severely restricted for most Cubans.

Remembering the hardships in the life of her father, who had been a sugar cane cutter, she talks about his emphasis on education for her and her siblings after the revolution, and how this instilled in her a desire to learn. Acknowledging that she is paid less “than some people who clean the streets” and “knows many people who have left,” she nevertheless talks with pride about her work. She explains how, despite all these difficulties (including the very real problems of transport), she still feels that what she does, working closely with families in the community where she lives, is part of a collective Cuban project working toward a better future.

In these examples, personal narratives of triumph over adversity in coming to work in the field of community genetics are articulated and expressed at the intersection with regional or national histories of revolutionary struggle. Although many were aware of and spoke about the contradictory contexts in which they lived and worked, there was nevertheless a strong sense of vocation among those I met. This commitment must be understood not simply in terms of blind allegiance to socialist credentials but the ways careers were entwined with a perception of genetic medicine as replete with future promise that had and could affect personal broader transformations in these (mostly) women's lives.

Like genetic medicine in other national contexts (see Stern Forthcoming[Bibr b37]), Cuban community genetics is overwhelmingly occupied by women. Here, the state policies that have facilitated women's entry into the workforce over the last 30 years, and that have led to the forging of careers within arenas such as community genetics, are mutually reinforced. The notion of a “revolutionary” gendered vocation was refracted not only through national *and* personal histories of triumph over adversity but also imagined and hoped for futures in Cuban science and health. As women who were still often also engaged in the daily *lucha* (struggle) for basic necessities for their families, it was a commitment that invested a great deal in the hoped-for personal as much as scientific transformations that a cutting-edge and modern field of health care such as community genetics seemed to offer.^8^

## Caring for the Health of the Population: Making Sense of State Paternalism and the Role of Sentiment

A notion of “care of the data” has served in the work of Mike Fortun as a shorthand for thinking about the agency and activities of scientists in the management, investigation, and presentation of complex “postgenomic” knowledge in the quite different context of Iceland. As Fortun puts it: “Understanding how scientists care for the data is a way to understand how society, culture and political-economy inhabit scientific practice and shape scientists’ own conceptions of themselves as agents of ethics and historical change” (http://mfortun.org/?page_id=72). Understanding what kind of agencies come to constitute care of the data in Cuban community genetics also demands taking account of the situated complexities in the work of these professionals.

Most of the research I did working with and alongside community genetic practitioners involved collecting family histories and discussing health beliefs about breast cancer with women in their homes. However, day-to-day work in community genetics revolves around the rigorous collection and collation of data related to neo- and prenatal screening programs. Here, the blood spot test, or *tamizaje*, is the central procedure by which public and population health care is managed and recorded. Tests are routinely made for over 10 different conditions. One community genetic doctor claimed that her regional center dealt with 30% of all pregnancies in the region, and an estimated 3,000 patients annually. At some of the weekly meetings of the genetics teams that I participated in, the importance of accurately recording and swiftly sending the results of genetic testing to clinics in Havana was routinely emphasized.

On the one hand, such activities point to the centralized control of results and data related to screening, highlighting the way that genetic medicine, like infant and maternal health in Cuba (Kath 2010), is part of the state management of health. However, when those I met described or demonstrated the work they did, they always stressed the importance of getting a good sample or taking care in completing the tamizaje test, explaining that knowing how the information was stored and utilized was part of the *collective* health interventions. Although the paternalism of the health care system was acknowledged, those I met routinely underlined the importance and value of the *community* health care provision they offered. This was particularly so in the eastern part of the country, where public health resources prior to state socialism had been scarce or nonexistent.^9^ Although ethical concerns existed for these health professionals, particularly related to issues of consent, the notion that community genetics was “leading the way” in addressing these ethical challenges mitigated these worries (Gibbon 2009).

One of the ways concerns about state paternalism were routinely assuaged by these professionals was by emphasizing work undertaken with children and adults with learning difficulties. One formative research project that had heralded the expansion of community genetics in 2000 was a study of the population prevalence of “major disabilities and mental retardation” across the country (Teruel 2009:11). As a result of this study, genetic professionals were now involved in local communities alongside social workers, helping affected families and individuals. Such activities, as the congress event discussed at the start of this article illustrated, showcased the humanitarian work of community genetics. This was reflected during the course of my fieldwork when community geneticists routinely chose to highlight these aspects of their work. For instance, on José Martí Day^10^ in a central province of the country, I was taken to the local center for children with learning disabilities to see their performance in celebration of the famous Cuban poet. As we watched a traditional dance performance by various groups of children and listened to a child's recital of one of Martí's poems, the geneticist I was with whispered that it was for this reason that she worked in community genetics, adding that it was “for love.”

Care of the data in the work of Cuban genetics, then, is firmly oriented to a focus on the collective health of the population and the community. Yet, although being informed by a background context in which state paternalism is present, daily working practices are also forged in the context of humanitarian concerns in which the mobilization of sentiment works to sustain and make evident the ethical goals of state socialist public health.

There is another important register in which the work of community genetics must be situated and understood that now forms a crucial part of public health provision in Cuba and that sits alongside and to some extent makes more challenging efforts to constitute its ethical parameters. As I explore below, this relates to the changing local economies of public health now operating in Cuba.

## Local Public Health Economies in Cuba

Examining the changing field of public health in Cuba in the wake of the collapse of the Soviet subsidies in the 1980s, the work of Sean Brotherton (2005, 2008) has illustrated how this is now in part constituted by the work of “socios”—where hard-to-get consumer goods and services are exchanged, including medical services and medications. Although evoking older social economies of patronage and “clientship” that have long formed part of the warp and woof of socialist societies (Verdery 2002), Brotherton sees these as part of an emerging entrepreneurial ethos that paradoxically now sustains public health (2008).

The work of Elise Andaya, examining how an economic climate of shortage affects the changing relations between health practitioners, state, and publics in reproductive medicine, sheds further light on these dynamics. Showing that “the gift” remains a central metaphor of Cuban medical practice that continues to bind health professionals, the state, and publics in a commitment to social and collective health, Andaya points to a new anxiety about its meaning and the moral status of doctors in Cuba's changing economic and political climate (2010: 357).

These issues were evident during field research as I moved in and between homes and locales to complete the questionnaires. The seemingly casual and spontaneous offers by health workers to help with *el problemo genético* if it ever arose in exchange for small gestures of assistance were frequent. Such exchanges could also be constituted by more elaborate gestures or gifts.^11^ These activities illustrate the way that community genetics is also part of local economies of exchange in public health in Cuba, where financial need, commitment to collective health, and the ethic or morality of “the gift of health” dynamically interact. They underline the limits of homogeneous readings of Cuban public health, including community genetics, as only encompassed by a culture of state paternalism.

Yet the reciprocities that collectively constitute a commitment to care in community genetics, particularly in emergent fields of genetic medicine such as cancer genetics, are not always so easily fulfilled, particularly when this is subject to rupture by very real resource limit as well as the ideological otherness and economic impossibility of pursuing genomics as personal medicine.

## The (Absent) Space of Breast Cancer Genetics: Equity, Technology, and the Problem of Personal Medicine

The collection of family history data for late onset adult conditions such as breast cancer, have, as previously mentioned, recently begun to form part of the work of community genetic practitioners. During field research, recording family history was linked to the questionnaire study investigating health beliefs about the causes of breast cancer.

Members of local communities who were invited to participate in the questionnaire study were certainly not surprised to also be asked about their family medical history. There was, in fact, often an easy exchange of information about the health of the extended family in the discussions that took place in the homes of those who participated in the questionnaire study. The longstanding system of Cuban family medicine ensures that the exchange of family history information is an expected and normalized aspect of public/professional health care dynamics.

However, these seemingly easy exchanges were also belied by a lack of other aspects of health care. That is, Cuban community genetic practitioners’ abilities to act on family history information related to conditions such as breast cancer are drastically hampered by a shortage of resources. The focus on genetic risk related to breast cancer through the collection of family trees (rather than genetic testing) not only highlights this gap but could also reveal other, more urgent shortcomings in the monitoring and treatment of cancer. Some of these difficulties emerged in a meeting recalled in the field notes below that took place in a family home while collecting questionnaire data with two genetic health practitioners and a woman who had only recently finished her treatment for breast cancer.

## Excerpt 3

We are sitting talking over a strong black sweet coffee with the woman and her husband who had entered the room and sat listening to our discussion. Talking about whether she has heard of genetic factors being involved in breast cancer, she mentions hearing something about *el pruebo para los genes* [a genetic test]. This prompts further questions about other interventions, which, because of cost and lack of availability in her local hospital, she wasn't able to have. This includes a test she's heard about that would have meant a “better” treatment being given to her.

The discussion becomes more heated after her husband launches into something of a monologue about the difficulties of living in Cuba. He recounts the challenge of getting to the hospital every day for treatment and the daily struggle to hunt for good food to feed his sick wife. It's a love-filled diatribe that silences the room and brings starkly into view for everyone (including the genetic professionals) the challenges in Cuba of dealing with diseases such as cancer. The geneticists acknowledge these difficulties, commenting on the challenges that all patients with cancer face. As we leave, the couple, although clearly frustrated by the inadequacies of the Cuban system, are nevertheless warm toward us and before leaving hand both geneticists (and myself) flowers. While it is a gesture that reflects the moral importance of reciprocity in the local economy of public health, it is a gift that all present knew could not in this case be easily honored given the very real resource limits not only in relation to procedures such as genetic testing but also more basic health care.

These interactions illustrate how a lack of resources in Cuba poses particular difficulties for the treatment of complex diseases such as cancer, which routinely require screening and monitoring as well as often costly treatment interventions. This situation also creates discomfort for genetic health practitioners in their efforts to incorporate risk assessment for conditions such as breast cancer within the broad panoply of community genetics. In this light, attending to family history, as part of an effort to intervene and engage with collective health, is undermined by the lack of economic resources and technological means fully to realize not only the future promise of genomics but provide a context in which the basic treatment of diseases such as cancer can be met for the majority. As one commentator put it: “Figuring in the equity factor**”** in relation to new health technologies in Cuba is hugely problematic (Medicc Review 2009).

It is perhaps not surprising that in this arena, the BRCA genes, the two inherited susceptibility genes linked to an increased risk of breast cancer and associated with transformations in genetic medicine in Euro-American societies in the last 10 years, constitute something of an absent space in Cuba. It is not only that routine genetic testing is impossible due to cost and lack of technological capacity, but also that there is doubt about the utility of identifying those at genetic risk. Yet, although many genetic professionals said as much, pointing to the limited treatment options for those at increased risk, this did not undermine the work of community genetics for them. In fact, in some cases the difficulties these professionals encountered in pursuing genetic medicine appeared to reinvigorate their commitment to a socialist public health ethos.

For a few practitioners, being able to counter the cultural stigma of cancer or the negative experience of having the disease in the family was sufficient impetus for the collection of family history. The community basis of genetic health care in Cuba necessarily has to take account of this lived context, attending and responding (where possible) to the social and psychological repercussions of disease as this plays out in the wider family. Attending to this broader social context might also mean being attentive to nongenetic factors.^12^

Interest in orienting Cuban community genetics in ways that fit broader social goals of public health was made evident in an exchange that happened one afternoon with a group of genetic practitioners after a weekly philosophy class that had been provided locally as part of their training.

## Excerpt 4

At the end of an intensive 3 weeks of interviewing in different regions of a central province, I am invited to attend the last philosophy class of the term for the community genetic professionals, which is being given by a local university professor. Nearly half the team are there when I arrive, busy scribbling notes. The lecture entitled “*Problem sociales de la ciencia e technología*” [Social Problems in Science and Technology] starts with a rhetorical discussion of “civilization” with criteria such as “progress” and “development” proposed and defined by the lecturer. Attention then turns to the “problems” of technology as defined by Marx. An illustrative example is given by the lecturer of the technologies that were used in Cuba in the 19th century to extract sugar cane and exploit the African slave population. The solution according to the lecturer is to form new relations of production with *socialismo* as the apex of a system of social progress where *el hombre vivir en un lugar donde no hay contradiciones entre los productors y métodos de productos* [humans can live in a place where there are no contradictions between producers and the methods of production]. In this, *el papel de la ciencia y technológica* [role of science and technology] is linked to *solidaridad, collectivismo, “el hombre nuevo,” maxísimo de los valores* [community, collectivism, “the new man”^13^ and ethical values]; all goals that, it is said, community genetics has at its center.

As we walk down the hill to lunch after, the themes of the lecture seem replete with contradictions. These are more apparent when the tourist buses zoom past to destinations Cubans no longer visit and that swell an industry that feeds growing inequities within Cuba society. There is initially little talk of the lecture itself. Instead discussion centers on the ostentatious gold jewelry that one doctor had recently acquired after her husband had returned from Venezeula following a medical mission there. The jealous admiration from others in the group that she is now able to afford such things seems to heighten further the contradictory subject of the lecture, given the implicit acknowledgment in these exchanges of how “humanist” medical missions have become part of the way that doctors and medical professions can earn extra money and also display their wealth.^14^

With some prompting, discussion does turn to the lecture. Starting with a recognition that the lesson had been a little bit more political than philosophical, Claudia chooses to reemphasize the message of the class that “community genetics is working for society.” “Science in Cuba,” she says, “has always been different … you see we're not just trying to help just one woman with breast cancer here, because science in Cuba is for everyone.” She then enthusiastically talks about programs of health awareness for breast cancer that she is planning to help develop in different parts of the province, recognizing the need for this following the collection of questionnaire data and the resulting discovery that many women were fearful or lacked knowledge of the disease.^15^

Claudia's response provides a striking indication of how the problematic promise of predictive or personal medicine was repositioned within the goals of socialist health care. Although contradictions and emerging moral complexities abound in the lives of these health professionals, commitment to the collective well-being of patients and publics is both sustained and sought. In this instance, the current technological and financial impossibility of developing personal genetic medicine in Cuba appears to reinforce the broader collective goals of public health, here articulated in terms of health promotion and raising awareness of disease. This brings the values of personal medicine rooted in Western individualism into critical question, showing how its parameters do not simply or easily fit the Cuban model of public health while also sustaining the wider ethic of collective community health.

## Conclusion

Drawing on ethnographic research in Cuba with community genetic professionals, this article has explored the intersections between genomics and public health, illustrating the diverse ways that science, sentiment, and particular kinds of biopolitical rationalities inform the emergence of this medical speciality. It points to the highly specific ways that global and globalizing health agendas can be diversely translated and operationalized at the local level. In doing so, it contributes to a growing body of work that questions the assumption that individualizing neoliberal identities are a necessary prerequisite to or homogeneously reproduced and sustained by the expansion and translation of genomics.

On the one hand, the Cuban material highlights the urgent need to attend to the way continuities in public health provision, including longstanding state involvement, can be formative in the development and expansion of genetic medicine. On the other hand, we see in the narratives and practices of health professionals the way “dense realms of sentiment” (Gammeltoft 2008:571) and specific ethical or humanitarian orientations constitute, limit, and circumscribe the practice of Cuban community genetic medicine. Although the figure of the self-sacrificing doctor is a discernible feature of both public rhetoric and personal narratives of health professionals, involvement in this field of health care is also read (by mostly female practitioners) as part of a modern and modernizing, forward-looking medical practice simultaneously rooted in revolutionary values and goals.

This duality reflects the multiple registers in which Cuban community genetics is being reproduced. That is, while a state socialist ethos of collective health seeps into daily practice, the work of community genetics, like other aspects of public health in Cuba, is also sustained by local economies of exchange that have come to constitute and maintain the gift of health. We also see the challenges of fledgling efforts to attend to diseases such as cancer as part of community genetics, given a lack of state resources to treat them or monitor those at risk of disease. Yet, these difficulties can also reinvigorate the goals of collective public health, situating the problem of personal medicine as an implicit critique of Western capitalist values.

The expansion of community genetics as part of a public health endeavor in Cuba can therefore not simply be seen as an extension of state paternalism any more than it can be read as part of a globalizing neoliberal health agenda. The registers at work are revealed here as multiple not singular, raising important questions about how questions of technology, power, and subjectivity might be understood and theorized in emerging transnational fields of medicine such as genomics. It suggests that detailed ethnographic exploration of the differences and links between “neoliberalism as exception and exceptions to neoliberalism” (Ong 2006:3) in these and other regions where global interactions influence in highly specific ways the governance and operation of biopolitical processes at the local level remains a vital task for medical anthropologists. 

In a recent publication, the director of the program of Cuban Community Genetics commenting on the global spread of genomics stated that personal medicine was “beyond the reach of the vast majority of individuals,” emphasizing that “community genetics in Cuba is developing in ways that give weight to both biological and social factors” (Teruel 2009:13). In an era in which both the stability and boundary of the gene have been and continue to be questioned, linked to increasing scientific complexity, and where gene/environment interactions are becoming an important terrain for understanding and acting on genomic health, such statements serve a dual purpose. They simultaneously articulate the modern and future scope of Cuban science and medicine *and* offer a critique of the West's individualism and biomedical reductionism, echoing a quest for “social medicine” that has a long history not only in state socialist Cuba but across Latin America (Porter 2006). The shifting terrain of post-genomic knowledge and technology raises new yet mainly still elusive possibilities for intervening in the health of individuals, but it remains to be seen whether and in what way alternative national models, such as Cuban community genetics, can participate and intervene in the global articulation of genomics as public health.

## Footnotes

1 Although the term “community genetics” emerged separately within an evolutionary biology setting (Ten Kate 1998), the term was first used in a medical context by WHO in 1987 to describe the provision of clinical genetics directly to the population (Modell 1992). Strict definitions have been subject to debate, especially as the term is closely linked to the notion of public health genetics. The approach is nevertheless characterized by an effort to bring clinical genetics to underdeveloped and developing countries and to addressing the well-being of the individual within society. It is a phenomenon that can be identified across several national contexts (see Knoppers and Brand 2009; Raz 2010).

2 Health workers, along with biotechnologies and pharmacy products, comprised 37% of the total export market in 2007 (see Andaya 2010).

3 It is important to note the recent political shifts in the time since the completion of fieldwork, which has seen the decision to allow individual Cubans to own property and to start their own businesses.

4 Published data in 2001 suggested a ratio of doctors to patients 1:175 (MINSAP 2001). These ratios are likely to be significantly less in the current context, with many doctors either having left the country or sent on medical missions (Andaya 2010).

5 Referral to genetic practitioners reflects this dual organization of health care. In other words, although patients may have been referred by their family doctor to a genetic practitioner, the close proximity between many health professionals and families (often literally living in close proximity to each other) can lead to more informal referral arrangements.

6 According to the Cuban National Cancer Registry, over 2,000 cases of breast cancer are diagnosed annually with an incidence of 40 per 100,000 inhabitants (Galán et al. 2009).

7 This comment identifies the region as both a site for the Cuban struggle for independence against Spain in the 19th century and key revolutionary struggles in the 1950s.

8 Women have been central to the goals of the Cuban revolution, particularly in the field of health (see Rosendhal 1997; Smith and Padula 1996).

9 See Rosendahl (1997) for further discussion of these regional differences.

10 *José Martí* is a powerfully resonant historically significant 19th-century figure seen as a symbol of Cuba's fight for independence against Spain.

11 These exchanges occurred on a number of occasions during the course of fieldwork. See Gibbon (2009:139) for a discussion of one such event.

12 See Gibbon (2009, 2010) for an examination of the way a range of risk factors that did not implicate personhood and individual responsibility were articulated by those completing the questionnaire.

13 The idea of *el hombre nuevo* is closely linked to older state discourse about how citizens and particularly medical professionals should reject material goods and self-interested concerns and instead turn their attention to the collective success of the socialist revolution (see Andaya 2010:362).

14 Medical missions to other countries by Cuban doctors have long been part of the program of socialism linked closely to the ideology of self-sacrifice (Feinsilver 1993). However, recent missions to Venezuela in return for oil have highlighted the fact that this enables doctors to earn extra money, generating uncertainty about the meaning of the Cuban gift of health (see Andaya 2010:369).

15 This ethnographic illustration has been discussed briefly in a previous article (see Gibbon 2009).
